# Serum C‐reactive protein greater than 75 mg/dL as an early available laboratory predictor of severe COVID‐19: A systematic review

**DOI:** 10.1002/iid3.1130

**Published:** 2023-12-28

**Authors:** Pershang Nazemi, SeyedAhmad SeyedAlinaghi, Ayein Azarnoush, Avin Mabadi, Arezoo Salami Khaneshan, Mohammadreza Salehi

**Affiliations:** ^1^ Department of Infectious Diseases, Imam Khomeini and Yas Hospital Complex Tehran University of Medical Sciences Tehran Iran; ^2^ Iranian Research Center for HIV/AIDS, Iranian Institute for Reduction of High‐Risk Behaviors Tehran University of Medical Sciences Tehran Iran; ^3^ Medical School Alborz University of Medical Sciences Karaj Iran; ^4^ Medical School Iran University of Medical Sciences Tehran Iran; ^5^ Department of Infectious Diseases, Imam Khomeini Hospital Complex Tehran University of Medical Sciences Tehran Iran

**Keywords:** COVID‐19, C‐reactive protein, inflammation, prognosis

## Abstract

**Introduction:**

Severe COVID‐19 management is still challenging. Having a laboratory factor to predict the severity of a patient's condition can be very useful in how to approach each patient. There have been studies concentrating on the correlation between serum C‐reactive protein (CRP) level and COVID‐19 severity but we aim to reach a threshold for CRP in disease severity determination.

**Methods:**

We conducted a thorough search on PubMed, Web of Science and Google Scholar databases from early 2019 to October 2021, and 323 studies were assessed for eligibility in three phases. We used the Newcastle‐Ottawa Scale to examine the validity of the studies. The *t*‐test was applied for the CRP level cutoffs.

**Results:**

Eventually, 11 articles and 1615 patients were included in this systematic review. Our analysis evaluated combined mean, median, and standard deviation of severe patients' CRP to be respectively 73.37, 53.80, and 47.936. Based on the combined mean, 75 mg/dL was suggested as an initial threshold for baseline CRP in hospitalized patients for developing severe conditions.

**Conclusion:**

This study recommends that COVID‐19 patients with on‐admission serum CRP levels of 75 mg/dL and more are likely associated with severe conditions. Thus, anti‐inflammatory agents and further following may be helpful in these patients.

## Graphical abstract

Having a laboratory factor to predict the severity of every COVID‐19 patient can be very useful in how to approach each patient. By reviewing 11 articles and 1615 patients, baseline serum CRP of 75 mg/dL in hospitalized patients was suggested as an early predictor for developing severe conditions.

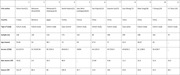

## 1 INTRODUCTION

The novel coronavirus pandemic emerging in China in January 2020, is the defining global health crisis of the moment and the greatest threat we have faced during this century.^1^, ^2^ Globally, as of 21 April 2022, there have been 505,035,185 confirmed cases of COVID‐19, including 6,210,719 deaths, reported by World Health Organization (WHO).^3^

The clinical manifestations of COVID‐19 disease are heterogeneous, including two forms severe and nonsevere. Therefore, COVID‐19 patients' management is adapted to the severity of the clinical situation. According to recent experiences, the majority of infected people are asymptomatic or have mild symptoms and can recover without medical intervention, whereas a small number of cases need to intensive care unit (ICU) care and mechanical ventilation.^4^, ^5^ Progression of the disease to a severe or critical clinical stage, significantly increases the risk of poor outcomes and mortality.^4^, ^6^, ^7^

Severe or life‐threatening COVID‐19 is characterized as a cytokine release syndrome (CRS), which is induced by a high‐mortality cytokine storm.^8^, ^9^ Early prediction of the infection severity can be effective in appropriate and necessary utilization of the healthcare resources.^10^ C‐reactive protein is the prototypical acute phase serum protein which can rise rapidly in response to inflammation, infection and tissue damage.^11^ The WHO Ordinal Scale for severity predicts mortality based on the clinical severity index, which has some limitations, such as not considering CRP.^12^ Therefore, this study aims to determine the association between the clinical severity of the disease and CRP levels upon admission.

## 2 MATERIAL AND METHODS

We systematically reviewed the studies reporting the relationship between early serum CRP level and COVID‐19 severity and outcome from the beginning of 2019 to the end of October 2021.

### 2.1 Search strategy

A systematic search was conducted on PubMed, Web of Science and Google Scholar from early 2019 to October 2021 using the keywords included:

A. “COVID‐19,” “novel coronavirus,” “SARS‐CoV‐2,” “*coronavirus* disease 2019.”
B. “CRP,” “C‐reactive protein,” “inflammatory Markers.”
C. “Outcome,” “prognosis,” “mild,” “moderate,” “severe,” “mortality.”

### 2.2 Selection criteria

The study implemented a comprehensive three‐phase screening process. Initially, two researchers scrutinized studies based on their titles and abstracts, excluding those meeting any of the following criteria:

- Duplicates.
- Non‐English language.
- Nonpeer‐reviewed status.
- Focus on children, pregnant women, or patients with underlying diseases.

Moving to the next phase, two additional researchers thoroughly examined the full texts of the remaining articles to assess their alignment with the following inclusion criteria:

- Original articles.
- Patients diagnosed with COVID‐19 and exhibiting a SARS‐CoV‐2 RNA‐positive result.
- Patients segregated into ICU and non‐ICU Groups, survivor, and nonsurvivor groups, or severe and nonsevere groups.
- Availability of CRP level data.
- Reported CRP values measured within the first day of hospitalization.

Finally, studies meeting the subsequent conditions were excluded:

- Sample size below 60.
- Reviews, case reports, and conference papers.
- Lack of clear diagnostic criteria for COVID‐19.
- Reporting outlier data.

### 2.3 Data extraction

Each of the two researchers who performed the literature search extracted specific data from eligible studies independently. The extracted variables included: type of study, country, sample size, mean age of patients, gender (female/male ratio), and C‐reactive protein (CRP) data.

In the reviewed literature different terminologies were used in the literature to report the outcomes. The severity of the patients' conditions was based on the World Health Organization categorization.^13^ In the current study, “severe,” “nonsurvivor” or “critical” groups are labeled as “severe” group. Similarly, “nonsevere,” “survivor,” and “ordinary” patients are categorized in the “nonsevere” group. The CRP data is reported and analyzed in severe group to determine predictability of serum CRP as a predictor of severe disease.

### 2.4 Quality/risk of bias assessment

The Newcastle‐Ottawa Scale (NOS) is an ongoing collaboration between the Universities of Newcastle, Australia and Ottawa, Canada. It was developed to assess the quality of nonrandomised studies with its design, content and ease of use directed to the task of incorporating the quality assessments in the interpretation of meta‐analytic results.^14^

We used the NOS to examine the validity of the studies. As shown in Table 1, all of the included literature scored 6 and higher.

**Table 1:** Quality assessment of manuscripts using the Newcastle‐Ottawa Scale.

First author	Selection (Out of 4)	Comparability (Out of 2)	Exposure/Outcome (Out of 3)	Total score (out of 9)
Ethem Acar^15^	**	*	***	6
Maryame Ahnach^16^	****	**	**	8
Mohamed El‐ shabrawy^10^	***	**	**	7
Semih Kalyon^17^	***	**	**	7
Jean‐Rémi Lavillegrand^18^	***	*	**	6
Yeu‐Ping liu^19^	****	**	**	8
Xiaomin luo^20^	***	**	***	8
Guyi Wang^21^	***	**	***	8
Miao Yang^22^	****	**	**	8
F Zheng^23^	**	**	**	6
Y‐Z Zhou^24^	***	**	**	7

### 2.5 Statistical analysis

All data was gathered in a Microsoft Excel worksheet and analysis was performed using SPSS version 26. 40, 50, 60, 70, and 75 were examined as possible CRP level cutoffs for predicting severity. For each possible threshold, *p* value was measured using the *t*‐test. *p* value ≤ .05 considered as a significant level.

## 3 RESULTS

**Figure 1:**
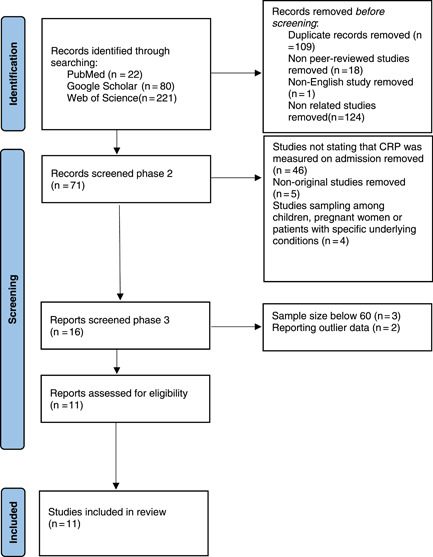
PRISMA flow diagram of selection process.

In total, 323 documents were identified using the mentioned search strategy. During the initial review, 109 duplicates, 18 nonpeer‐reviewed studies, 1 non‐English study, and 124 nonrelated studies were excluded. In phase two of the screening, 71 articles were retrieved for further evaluation. During the full‐text assessment of the articles, those not mentioning on‐admission CRP measurement, or were sampled among pregnant women, children, or patients with underlying diseases, and nonoriginal studies were excluded. The third screening phase excluded 5 more articles due to the narrow sample size and reporting outlier data (Figure 1/PRISMA flowchart). As shown in Table 1, the 11 remaining reports were qualitatively assessed using the NOS. All of the above had overall acceptable quality, scoring 6 and above in NOS; Among them 6 were conducted in China,^19^, ^20^, ^21^, ^22^, ^23^, ^24^ 2 in Turkey^15^, ^17^ and 1 study in each of the following countries: Morocco,^16^ Egypt,^10^ and France.^18^ We summarized the process of study identification and selection in Figure 1.

Table 2 describes the characteristics of the patients included in this study. A total of 1615 cases were categorized into severe and nonsevere subgroups. We aimed in this study to establish a threshold for on‐admission CRP which can be used as an early predictor for the COVID‐19 severity.

**Table 2:** CRP levels in severe and nonsevere patients.

	1	2	3	4	5
**First author**	Ethem Acar^15^	Maryame Ahnach^16^	Mohamed El‐shabrawy^10^	Semih Kalyon^17^	Jean‐Rémi Lavillegrand^18^
**Country**	Turkey	Morocco	Egypt	Turkey	France
**Type of study**	Cohort study	Cohort study	Cohort study	Case‐control	Cohort study
**Sample size**	148	145	116	175	101
**Age (mean)**	59.45	50	38.7	73	59
**Gender (F/M)**	62.2/37.8	51.72/48.28	45.7/54.3	58.9/41.1	18/82
**Non severe CRP**	78.62	3.4	20.5	40.9	_
**Severe CRP**	192.27	86.4	46	105.9	45.7

	6	7	8	9	10	11
**First author**	Yeu‐Ping liu^19^	Xiaomin luo^25^	Guyi Wang^21^	Miao Yang^22^	F Zheng^23^	Y‐Z Zhou^24^
**Country**	China	China	China	China	China	China
**Type of study**	Cohort study	Cohort study	Case series	Cohort study	Cohort study	Cohort study
**Sample size**	84	298	209	108	161	70
**Age (mean)**	47.9	57	42.9	50.67	45	_
**Gender (F/M)**	44.1/55.9	49.7/50.3	49.8/50.2	61.1/38.9	50.3/49.7	_
**Non severe CRP**	37.69	9.65	12.1	15.4	15.4	10.9
**Severe CRP**	12.9	100	43.8	53.8	52.2	68.1

Initially, the combined mean, median, and standard deviation of on‐admission CRP were measured in the severe subgroup as followed: 73.37 combined mean, 47.94 standard deviation (SD) and 53.80 median. Next, for each probable threshold, percentage of patients who aggravated towards severity with CRP levels above and below the threshold were compared and analyzed. For assumed threshold 75, 51.25% of patients with CRP levels of 75 and above deteriorated towards severe conditions. on the other hand, only 19.14% of patients with CRP levels below 75 exacerbated towards severity. For the mentioned cutoff P‐value was measured as 0.015 and clinical significance was set at *p* = .05. Therefore, baseline CRP level of 75 and above can be assumed as a reliable predictor for the severe disease. The sensitivity and specificity values for the mentioned cutoff are 72.8% and 100% respectively.

## 4 DISCUSSION

This study provides the CRP serum level threshold for prediction of COVID‐19 severity by reviewing the literature. In our review, there are more than 100 articles on CRP and COVID‐19 severity, of which 11 studies finally met the inclusion criteria of the systematic review and were included in this study. It seems that this inflammatory index is one of the most important and common laboratory markers that has been taken into consideration in different regions of the world to predict the COVID‐19 severity.^26^, ^27^, ^28^, ^29^ Although, in our investigation, interestingly, more than 50% of the studies were from China.

C‐reactive protein is an acute‐phase inflammatory protein that was first discovered in 1930 by Tillet and Francis.^30^ Induction of CRP gene transcription occurs mainly in hepatocytes in response to increased levels of inflammatory cytokines, particularly interleukin‐6 (IL‐6).^31^ CRP is considered as a relatively sensitive biomarker in infectious and noninfectious inflammation, and during acute inflammatory responses, CRP levels increase rapidly.^32^, ^33^ The average serum CRP level in a healthy Caucasian is about 0.8 mg/L, but this baseline level can vary widely among individuals due to other factors, including age, gender, blood pressure, hyperlipidemia, and polymorphisms in the CRP gene.^34^, ^35^ In this review, very high serum concentrations of CRP (near to 1000‐fold) were found as the predictor for severe COVID‐19 condition, which were previously seen in some bacterial sepsis.^36^

Severe pulmonary involvement and mortality in COVID‐19 patients are associated with immune and inflammatory system dysregulation.^37^, ^38^, ^39^ Several studies have reported cellular and molecular changes related to immunity include lymphopenia, neutrophilia, inflammatory cytokines, IL‐6, TNF‐α granulocyte colony‐stimulating factor (G‐CSF) and monocyte chemoattractant protein‐1 (MCP1) in severe COVID‐19 patients.^40^, ^41^, ^42^, ^43^ It has been found that IL‐6 is the main inducer of CRP gene expression and IL‐1 enhances its effect, although IL‐6 is necessary for CRP gene induction, it is not sufficient to achieve this alone.^44^, ^45^ The cytokine storm in COVID‐19, which has been identified as an important cause of ARDS, mainly depends on IL‐6, and it seems that the relationship between CRP and interleukin‐6 can justify the use of serum CRP level in prophecy for the entry of patients into the cytokine release syndrome.^46^, ^47^ In our results, CRP 75 mg/dL and more is clearly associated with the clinical deterioration of patients, which is caused by the inflammatory storm in these patients.

Although there is a relationship between the clinical and radiological severity of COVID‐19 and the serum CRP level in various previous studies, the definitive cut‐off has not been clearly presented until now.^10^, ^26^, ^48^, ^49^, ^50^ One of the important topics in the management and treatment of patients with severe COVID‐19 is the time of prescription of anti‐inflammatory agents such as corticosteroids and IL‐6 antagonists for cytokine storm controlling.^51^, ^52^, ^53^ In various guidelines and studies, some respiratory, radiological and laboratory markers have been mentioned to express the severity of COVID‐19 and the initiation of anti‐inflammatory therapy, of which the most important and common index is the level of serum CRP.^54^, ^55^, ^56^ Within RECOVERY Collaborative Group, one of the inclusion criteria to receive tocilizumab or the standard of care was CRP ≥ 75 mg/L as a marker of systemic inflammation, which is interestingly consistent with the cutoff we obtained in our study.^57^

The strengths of the present study are that it has reviewed CRP markers in a significant number of studies of critical COVID‐19 patients that had a standard design. The most important limitations of this study is that access to the changes of this inflammatory factor was not based on the patients demographic.

## 5 CONCLUSION

Relying on the results of our analysis, the threshold of 75 mg/dL for on‐admission CRP levels of patients with COVID‐19 is prognostic. That means patients with CRP levels above 75 mg/dL on admission have a higher risk of contributing to severe COVID‐19. Having an early prognostic factor can guide us to a better approach to the patient. The sensitivity of 72.8% and specificity of 100% for the mentioned CRP cutoff suggest that if a patient's CRP level upon admission exceeds 75, it is highly indicative that they will deteriorate to severe conditions. To be able to use this conclusion in clinical practices meta‐analysis should be done.

## AUTHOR CONTRIBUTIONS

**Pershang Nazemi**: Visualization (lead); investigation (support); methodology (support); project administration (support); supervision (support); writing original draft (support); writing review and editing (support). **SeyedAhmad SeyedAlinaghi**: Data curation (lead); formal analysis (lead); validation (lead); methodology (support); resources (equal). **Ayein Azarnoush**: Formal analysis (support); investigation (lead); project administration (lead); resources (equal); methodology (support); visualization (support); writing original draft (lead); writing review and editing (lead). **Avin Mabadi**: Investigation (support); visualization (support); writing original draft (support); writing review and editing (support). **Arezoo Salami khaneshan**: Investigation (support); visualization (support). **Mohammadreza Salehi**: Conceptualization (lead); methodology (lead); supervision (lead); validation (support).

## CONFLICT OF INTEREST STATEMENT

The authors declare no conflict of interest.

## ETHICS STATEMENT

All analysis was based on previous published studies; thus, no ethical approval is required.

## Data Availability

The authors have nothing to report.
